# Verapamil Parameter- and Dose-Dependently Impairs Memory Consolidation in Open Field Habituation Task in Rats

**DOI:** 10.3389/fphar.2016.00539

**Published:** 2017-01-10

**Authors:** Natalija Popović, Verónica Giménez de Béjar, María Caballero-Bleda, Miroljub Popović

**Affiliations:** ^1^Department of Human Anatomy and Psychobiology, Faculty of Medicine, University of MurciaMurcia, Spain; ^2^Instituto Murciano de Investigación Biosanitaria, Virgen de la ArrixacaMurcia, Spain; ^3^Department of Neurology, Santa Lucía University General HospitalCartagena, Spain

**Keywords:** habituation, memory consolidation, open field, verapamil, rat

## Abstract

The purpose of the present study was to examine the effects of the phenylalkylamine class of the L-type voltage-dependent calcium channel antagonist, verapamil (1.0, 2.5, 5.0, or 10 mg/kg i.p.), administered immediately after the acquisition task, on memory consolidation of the open field habituation task, in male Wistar rats. On the 48 h retested trial, all tested parameters (ambulation in the side wall and in the central areas, number of rearing, time spent grooming and defecation rate) significantly decreased in the saline treated animals. A significant decrease of rearing was observed in all verapamil treated groups. On the retention day, the ambulation in the side wall and central areas significantly decreased in the animals treated with 1 mg/kg and 10 mg/kg of verapamil, while the time spent grooming and the defecation rate significantly decreased only in the group treated with 1 mg/kg of verapamil. According to the change ratio scores that correct the individual behavioral baseline differences during initial and final sessions, habituation deficit was found in animals treated with verapamil as follows: ambulation along the side wall area (1, 2.5, and 5 mg/kg), number of rearing (all used dose) and time spent grooming (2.5, 5, and 10 mg/kg). In conclusion, the present data suggest that the post-training administration of verapamil, parameter- and dose-dependently, impairs the habituation to a novel environment.

## Introduction

Three different subfamilies of calcium (Ca_v_) channels have, thus far, been defined and named Ca_v_1, Ca_v_2, and Ca_v_3 (Ertel et al., 2000; Catterall et al., 2005; Hofmann et al., 2014). The Ca_v_1 subfamily (Ca_v_l.l–Ca_v_l.4) includes channels which mediate L-type Ca^2+^currents, whereas the Ca_v_2 subfamily (Ca_v_2.1–Ca_v_2.3) comprises channels which originate P/Q-, N-, and R-type Ca^2+^currents, respectively. Lastly, the Ca_v_3 subfamily (Cav3.1–Cav3.3) includes channels which mediate T-type Ca^2+^ currents (Ertel et al., 2000; Catterall et al., 2005; Hofmann et al., 2014).

Molecular genetics approaches clearly suggest essential implication of the Ca_v_1.3 but not the Ca_v_1.2 subunit of the L-type voltage-gated calcium channels (LVGCCs), in the process of memory consolidation of contextual fear conditioning in mice (McKinney and Murphy, 2006; McKinney et al., 2008). However, the effects of several classes of LVGCC antagonists, such as dihydropyridines (e.g., nimodipine, nicardipine, and nifedipine), benzothiazepines (e.g., diltiazem), phenylalkylamines (e.g., verapamil), and diphenylalkylamines (e.g., flunarizine), on memory consolidation in young mice and rats, are controversial. It has been found that nimodipine does not affect the memory consolidation of rats tested in the simple learning association (Isaacson et al., 1989), as well as of mice tested in contextual fear conditioning (Suzuki et al., 2004). Calcium channel blockers that bind principally to dihydropyridine receptors (nimodipine, nifedipine, and amlodipine) do facilitate memory consolidation of mice tested in passive avoidance, linear maze, and elevated plus-maze tasks (Quartermain et al., 1993, 2001; Biala et al., 2013). On the other hand, diltiazem and flunarizine improve memory consolidation of mice tested in passive avoidance and linear maze (Quartermain et al., 1993, 2001) but not in the elevated plus-maze task (Biala et al., 2013). Studies in mice demonstrated that systemic post-training treatment with verapamil does not affect memory consolidation in the passive avoidance task (Quartermain et al., 1993; Masoudian et al., 2015), but improves retention in linear maze and elevated plus maze tasks (Biala et al., 2013). Noting the lack of data related to the effect of LVGCC antagonists on memory consolidation in rats, the aim of the present study was to evaluate the effects of verapamil post-training treatment on memory retention, in the open field habituation task.

## Materials and Methods

### Experimental Animals

Experiments were carried out on male Wistar rats, weighing 200–250 g. The animals were housed in standard Makrolon cages on sawdust bedding. They were kept in an air-conditioned room (20 ± 1°C), at 30% humidity and under a 12 h light/12 h dark cycle (lights on from 08:00 to 20:00 h). Food and tap water were available *ad libitum.* One week before the experimental procedure, the rats were handled daily for 5 min each. The behavioral tests were performed during the light period (16:00–20:00 h).

All procedures related to the animal maintenance and experimentation were in accordance with the European Communities Council Directive of November 24, 1986 (86/609/EEC) and the guidelines issued by the Spanish Ministry of Agriculture, Fishing and Feeding (Royal Decree 1201/2005 of October 21, 2005) and were approved by the Animal Ethics Committee of the University of Murcia. Efforts were made to minimize the number of animals used, as well as their suffering.

### Drugs

The saline solution of verapamil (Sigma, St. Louis, MO, USA) was administered intraperitoneally at the dose of 1, 2.5, 5, or 10 mg/kg. Control animals were treated with physiological saline at the dose of 1 ml/kg body weight. Eight animals were assigned in each tested group.

### Open Field Test

The open field test was performed in a square white plywood box (100 cm × 100 cm × 40 cm). The floor was divided into 25 (20 × 20 cm) squares. On day 1, the rats were initially placed at one of the four corners of the box and their behavior was monitored during 10 min. After that, the rats were removed from the open field, drug administered and returned to their home cage. Forty-eight hours later, the retention test was given. The open field test was performed under 300 lux light intensity and recorded using a video camera to enable subsequent evaluation. The apparatus was cleaned with 70% ethanol before each animal was tested. Eight animals were assigned in each tested group.

In the open field test, the *ambulation along the side wall area* (number of outer squares entered), the *ambulation in the central area* (number of inner squares entered), the *number of rearing* (standing on the hind legs, with or without contact with the sides of the arena), the *time spent frozen* (time that the animal spent immobile), the *time spent in grooming* (time that the animal spend licking, scratching or cleaning any part of its head or body) and the *defecation* (number of fecal boli deposited) were recorded. With the aim to correct the individual baseline differences in the studied parameters, we used a change ratio score to compare behavior during initial and final sessions. This score is calculated according to the Bolivar (2009) as follows: day 2 parameter value/(day 1 parameter value + day 2 parameter value). Thus, the change ratio score will approach 0.5 if no change in behavior has occurred, i.e., no habituation. If the ratio approaches 0, there is evidence of habituation. If the ratio approaches 1.0, the tested behavioral parameter has actually increased from the initial to the final time periods.

### Statistical Analysis

The statistical analysis was made using the SPSS 19.0 statistical package. The data were analyzed with the General Linear Model (GLM) repeated measures analysis and presented as mean ± standard error of the mean (SEM). If the GLM showed significant differences between groups, a *post hoc* analysis was performed. The group differences on the acquisition trial were analyzed by the two-tailed Student’s *t*-test for independent samples. The two-tailed Student’s *t*-test for paired-samples was used for comparison of the data between the acquisition and the retention trial. The habituation scores were analyzed by the one-way ANOVA test, followed by the two-tailed Student’s *t*-test for independent samples. Differences were considered statistically significant if *p* < 0.05.

## Results

### Open Field Test

**FIGURE 1:**
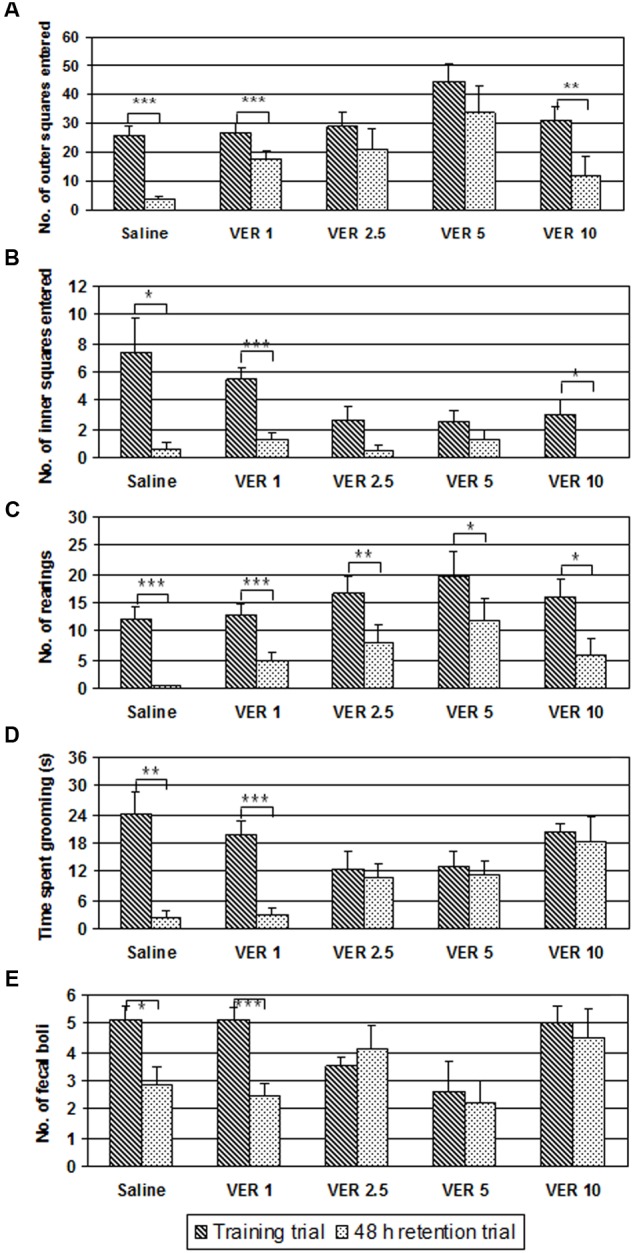
**Effect of 1, 2.5, 5, and 10 mg/kg of verapamil (VER-1, VER-2.5, VER-5, and VER-10, respectively), administered intraperitoneally, immediately after the acquisition trial, on ambulation in the side wall area (A)**, ambulation in the central area **(B)**, number of rearing **(C)**, time spent grooming **(D)**, and defecation **(E)**, in the open field habituation task. The data are presented as mean ± standard error of the mean (SEM). ^∗^*p* < 0.05, ^∗∗^*p* < 0.01, ^∗∗∗^*p* < 0.001 vs. acquisition trial.

Only one animal from the saline treated group and four animals from the verapamil treated groups displayed freezing behavior (data not showed). The rest of the data from the open field test are presented in the **Figure 1**.

The GLM repeated measure analysis showed a significant *effect of time* on ambulation in the side wall area (*F*_(1)_ = 47.577, *p* < 0.001), ambulation in the central area (*F*_(1)_ = 42.976, *p* < 0.001), number of rearing (*F*_(1)_ = 64.390, *p* < 0.001), time spent grooming (*F*_(1)_ = 19.682, *p* < 0.001), and defecation (*F*_(1)_ = 6.712, *p* < 0.05). There was significant *effect of group* on ambulation in the side wall area (*F*_(4)_ = 3.207, *p* < 0.05) but not on ambulation in the central area (*F*_(4)_ = 1.987, *p* > 0.05), number of rearing (*F*_(4)_ = 2.013, *p* > 0.05), time spent grooming (*F*_(4)_ = 2.006, *p* > 0.05), and defecation (*F*_(4)_ = 2.454, *p* > 0.05). There was significant *effect of interaction time × group* on ambulation in the central area (*F*_(4)_ = 3.261, *p* < 0.05) and on the time spent grooming (*F*_(2)_ = 4.785, *p* < 0.01) but not on ambulation in the side wall area (*F*_(4)_ = 2.128, *p* > 0.05), number of rearing (*F*_(4)_ = 0.372, *p* > 0.05), and defecation (*F*_(4)_ = 2.390, *p* > 0.05).

In the acquisition trial of the open field test, there were no significant differences between groups in the ambulation in the side wall area (*F*_(4)_ = 2.591, *p* > 0.05), ambulation in the central area (*F*_(4)_ = 2.542, *p* > 0.05), number of rearing (*F*_(4)_ = 1.061, *p* > 0.05), time spent grooming (*F*_(4)_ = 2.233, *p* > 0.05), and defecation rate (*F*_(4)_ = 2.604, *p* > 0.05). The two-tailed Student’s *t*-test for paired-samples showed that ambulation in the side wall (**Figure 1A**) and in the central (**Figure 1B**) areas, significantly decreased on the retention day, in the saline group (*t* = 6.677, *df* = 7, *p* < 0.001; *t* = 3.441, *df* = 7, *p* < 0.05, respectively) and in the animals treated with verapamil at the dose of 1 mg/kg (*t* = 5.105, *df* = 7, *p* < 0.001; *t* = 6.298, *df* = 7, *p* < 0.001, respectively) and 10 mg/kg (*t* = 3.486, *df* = 7, *p* < 0.01; *t* = 2.722, *df* = 7, *p* < 0.05, respectively) but not in the animals treated with verapamil at the dose of 2.5 (*t* = 2.004, *df* = 7, *p* > 0.05; *t* = 1.945, *df* = 7, *p* > 0.05, respectively) and 5 mg/kg (*t* = 1.708, *df* = 7, *p* > 0.05; *t* = 2.236, *df* = 7, *p* > 0.05, respectively). The two-tailed Student’s *t*-test for paired-samples showed that the number of rearing (*t* = 5.276, *df* = 7, *p* < 0.001; *t* = 5.420, *df* = 7, *p* < 0.001; *t* = 3.804, *df* = 7, *p* < 0.01; *t* = 2.396, *df* = 7, *p* < 0.05; *t* = 3.155, *df* = 7, *p* < 0.05) (**Figure 1C**) significantly decreased on the retention day in both the saline- and verapamil-treated (1, 2.5, 5, and 10 mg/kg) animals, respectively. On the retention day, the time spent grooming (**Figure 1D**) and the defecation rate (**Figure 1E**) significantly decreased in the groups treated with saline (*t* = 4.376, *df* = 7, *p* < 0.01; *t* = 3.000, *df* = 7, *p* < 0.05, respectively) and verapamil at the dose of 1 mg/kg (*t* = 6.120, *df* = 7, *p* < 0.001; *t* = 8.104, *df* = 7, *p* < 0.001, respectively) but not in animals treated with verapamil at the doses of 2.5 mg/kg (*t* = 0.501, *df* = 7, *p* > 0.05; *t* = -0.804, *df* = 7, *p* > 0.05, respectively), 5 mg/kg (*t* = 0.367, *df* = 7, *p* > 0.05; *t* = 0.351, *df* = 7, *p* > 0.05, respectively), and 10 mg/kg (*t* = 0.346, *df* = 7, *p* > 0.05; *t* = 0.408, *df* = 7, *p* > 0.05, respectively).

**FIGURE 2:**
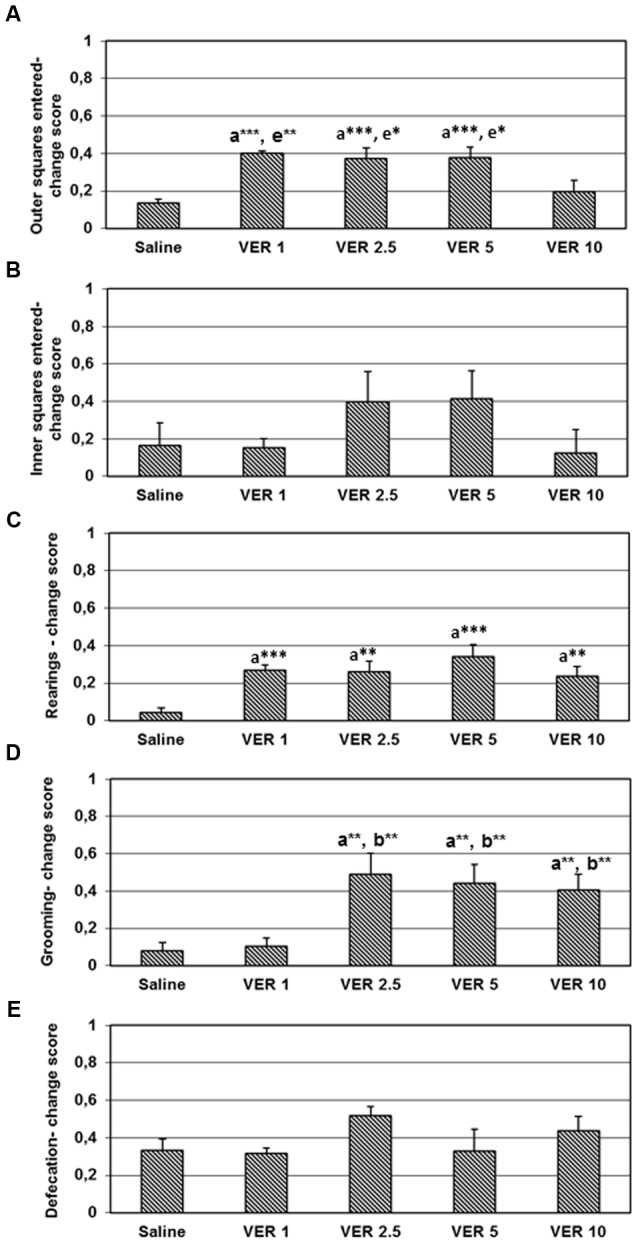
**Effect of 1, 2.5, 5, and 10 mg/kg of verapamil (VER-1, VER-2.5, VER-5, and VER-10, respectively), administered i.p. immediately after the acquisition trial, on habituation score in ambulation in the side wall area (A)**, ambulation in the central area **(B)**, number of rearing **(C)**, time spent grooming **(D)**, and defecation **(E)**, in the open field habituation task. The data are presented as mean ± SEM. ^∗∗^*p* < 0.01, ^∗∗∗^*p* < 0.001 vs. acquisition trial. ^a,b,e^ vs. saline, VER-1 or VER-10 groups, respectively.

The one-way ANOVA test revealed significant differences in the change ratio score for ambulation in the side wall area (*F*_(4)_ = 7.097, *p* < 0.001), number of rearing (*F*_(4)_ = 5.169, *p* < 0.01) and time spent grooming (*F*_(4)_ = 5.616, *p* < 0.001) but not in ambulation in the central area (*F*_(4)_ = 1.266, *p* > 0.05) and defecation rate (*F*_(4)_ = 1.539, *p* > 0.05). The *post hoc* analysis showed that the change ratio score for ambulation in the side wall area was significantly higher in animals treated with verapamil in doses of 1, 2.5, and 5 mg/kg than in those treated with saline (*t* = -10.104, *df* = 14, *p* < 0.001; *t* = -4.042, *df* = 14, *p* < 0.001; *t* = -4.091, *df* = 14, *p* < 0.001, respectively) or 10 mg/kg of verapamil (*t* = 3.242, *df* = 14, *p* < 0.01; *t* = -2.154, *df* = 14, *p* < 0.05; *t* = 2.195, *df* = 14, *p* < 0.05, respectively) (**Figure 2**). The change ratio score for the number of rearing was significantly higher in the animals treated with verapamil (1, 2.5, 5, and 10 mg/kg), compared to those treated with saline (*t* = -6.216, *df* = 14, *p* < 0.001; *t* = -3.501, *df* = 14, *p* < 0.01; *t* = -4.219, *df* = 14, *p* < 0.001; *t* = -3.349, *df* = 14, *p* < 0.01, respectively) (**Figure 2**). The animals treated with verapamil in doses of 2.5, 5, and 10 mg/kg have significantly higher change score for time spent grooming than the animals treated with saline (*t* = -3.307, *df* = 14, *p* < 0.01; *t* = -3.289, *df* = 14, *p* < 0.01; *t* = -3.431, *df* = 14, *p* < 0.01, respectively) and verapamil in dose of 1 mg/kg (*t* = -3.129, *df* = 14, *p* < 0.01; *t* = -3.092, *df* = 14, *p* < 0.01; *t* = -3.206, *df* = 14, *p* < 0.01, respectively) (**Figure 2**).

## Discussion

In the present study, we demonstrated that verapamil, dose- and parameter-depending, affects habituation in the open field test, one of the most elementary forms of non-associative hippocampal-dependent learning (Leussis and Bolivar, 2006). Although, the animals treated with verapamil (in all tested doses), significantly reduced the rearing component of the exploratory behavior on the retention trial, the level of habituation was impaired compared to the saline treated animals. In contrast, the middle-used doses of verapamil (2.5 and 5 mg/kg) did not significantly decrease the ambulation component of the exploratory behavior, indicating, on this parameter, the inverted U-shape dose-response curve of its action. However, the habituation score of ambulation in the side wall area (verapamil 1, 2.5, and 5 mg/kg) was significantly impaired, but not the habituation score of ambulation in the central area. Regarding to grooming and defecation, a significant decrease in those parameters was found only in animals treated with verapamil in the dose of 1 mg/kg. In spite of this, the habituation score of grooming was significantly lower in animals treated with verapamil (2.5, 5, and 10 mg/kg) compared to the saline treated animals. The present data support our previous findings that mechanisms of habituation are complex and that each component could be differently affected by the drugs (Popović, 2014).

It has been demonstrated that verapamil treatment at the doses of 1, 2, and 10 mg/kg, but not at the dose of 20 mg/kg, significantly improves the consolidation of spatial memory in the linear maze task in mice (Quartermain et al., 2001). Similarly, verapamil at the doses of 5 and 10 mg/kg, but not at the doses of 2.5 and 20 mg/kg, displays an enhancement effect on the consolidation of spatial memory in the elevated plus maze task in mice (Biala et al., 2013), resembling the inverted U-shape dose-response curve of its action. It has been suggested that the inverted U-shape dose-response curve of the verapamil action, could be due to modulation rather than to a complete blockade of LVGCCs (Biala et al., 2013).

The mechanism by which verapamil facilitates or impairs memory consolidation in different tasks, is still unclear. Although classified as L-type calcium channel blocker (being Ca_v_1.2 channels more sensitive than the Ca_v_1.3 ones), verapamil blocks Ca_v_2.1, Ca_v_2.2, Ca_v_2.3, and Ca_v_3.2 channels too (Ishibashi et al., 1995; Cai et al., 1997; Dobrev et al., 1999; Tarabova et al., 2007; Kuryshev et al., 2014). Given that the systemic administration of verapamil (range dose 1–10 mg/kg) does not affect the open field behavior (Rogério and Takahashi, 1990; Popović, 1997), the effect of verapamil on memory consolidation of the open field habituation, seems to be independent on the anxiety level. In this line, in Ca_v_1.3 knockout mice the open field behavior was not changed (Busquet et al., 2010), while only severe reduction in the Ca_V_1.2 activity, in the mouse forebrain, enhanced anxiety-like behaviors in open field (Lee et al., 2012). As far to our knowledge, there are no data analyzing the implication of these subunits in the consolidation of the open field habituation.

In view of the fact that verapamil can block large conductance calcium-activated potassium channels (BK channel) (Harper et al., 2001) and that habituation of the exploratory locomotor behavior in the open field is not affected in the BK knock-out mice (Typlt et al., 2013), it could be less probable that the effect of verapamil be partially due to the action on these channels. It is possible that verapamil, through its action as an antagonist of P-glycoprotein transporters or multidrug resistance proteins (Pajeva and Wiese, 2002), impairs memory consolidation in open field task, since P-glycoprotein knockout mice display lower level of habituation than the wild-type mice (Schoenfelder et al., 2012).

## Conclusion

As far to our knowledge, the present data represent the first evidence that post-training verapamil administration produces parameter- and dose-dependent impairment of habituation in the open field task in rats. Moreover, the present findings resembling the inverted U-shape dose-response curve of verapamil action.

## Ethics Statement

All procedures related to the animal maintenance and experimentation were in accordance with the European Communities Council Directive of November 24, 1986 (86/609/EEC) and the guidelines issued by the Spanish Ministry of Agriculture, Fishing and Feeding (Royal Decree 1201/2005 of October 21, 2005) and were approved by the Animal Ethics Committee of the University of Murcia. Efforts were made to minimize the number of animals used, as well as their suffering.

## Author Contributions

All authors (NP, VGdB, MC-B, and MP) contributed to the design of the study, wrote the protocol and managed the literature searches. Authors NP, VGdB, and MP performed the experiments and undertook the statistical analysis. All authors (NP, VGdB, MC-B, and MP) contributed to drafting the work and have approved the final manuscript.

## Conflict of Interest Statement

The authors declare that the research was conducted in the absence of any commercial or financial relationships that could be construed as a potential conflict of interest.
